# Kinetics of functional beta cell mass decay in a diphtheria toxin receptor mouse model of diabetes

**DOI:** 10.1038/s41598-017-12124-w

**Published:** 2017-09-29

**Authors:** Pim P. van Krieken, Andrea Dicker, Maria Eriksson, Pedro L. Herrera, Ulf Ahlgren, Per-Olof Berggren, Erwin Ilegems

**Affiliations:** 1 The Rolf Luft Research Center for Diabetes and Endocrinology, Karolinska Institutet, Stockholm, Sweden; 2 Umeå Centre for Molecular Medicine, Umeå University, Umeå, Sweden; 3 Department of Genetic Medicine and Development, University of Geneva Medical School, Geneva, Switzerland; 4 Diabetes Research Institute, Miller School of Medicine, University of Miami, Miami, USA; 5 Lee Kong Chian School of Medicine, Nanyang Technological University, Imperial College London, Novena Campus, Singapore, Singapore

## Abstract

Functional beta cell mass is an essential biomarker for the diagnosis and staging of diabetes. It has however proven technically challenging to study this parameter during diabetes progression. Here we have detailed the kinetics of the rapid decline in functional beta cell mass in the RIP-DTR mouse, a model of hyperglycemia resulting from diphtheria toxin induced beta cell ablation. A novel combination of imaging modalities was employed to study the pattern of beta cell destruction. Optical projection tomography of the pancreas and longitudinal *in vivo* confocal microscopy of islets transplanted into the anterior chamber of the eye allowed to investigate kinetics and tomographic location of beta cell mass decay in individual islets as well as at the entire islet population level. The correlation between beta cell mass and function was determined by complementary *in vivo* and *ex vivo* characterizations, demonstrating that beta cell function and glucose tolerance were impaired within the first two days following treatment when more than 50% of beta cell mass was remaining. Our results illustrate the importance of acquiring quantitative functional and morphological parameters to assess the functional status of the endocrine pancreas.

## Introduction

The pancreatic beta cells are essential for the maintenance of adequate blood glucose levels. In response to an increase in circulating glucose concentration these endocrine cells secrete insulin, a hormone required for proper glucose uptake from various organs in the body. An insufficient number of functional beta cells will lead to a disturbance in blood glucose homeostasis and thus to the development of diabetes. Both for diagnostic and prognostic reasons, it is therefore of utmost importance to be able to assess the functional status of pancreatic beta cells.

Beta cell mass (BCM), a collective term designating the entire population of beta cells in the pancreas, is commonly used as a biomarker for the function of the endocrine pancreas^1–3^. From partial pancreatectomy studies it is known that humans and rodents have a significant overcapacity in terms of potential to secrete insulin, being able to sustain normoglycemic levels with only 10–30% of the initial BCM remaining^4–8^. Yet, it is unclear whether BCM is an appropriate biomarker under all circumstances and for all species. In various rodent models of hyperglycemia the relationship between beta cell mass and function is indeed not fully established. While a strong reduction in beta cell mass generally correlates with hyperglycemia, for instance after chemical ablation of beta cells (i.e. streptozotocin or alloxan^9^) or in type 1 diabetes models (i.e. in the non-obese diabetic NOD mouse^10,11^ or the BioBreeding BB-rat^12,13^), an abnormally high number of beta cells can also be observed in the hyperglycemic leptin-deficient ob/ob mouse^14,15^. In humans, autopsy studies report substantial differences in the severity of beta cell loss in both type 1 and type 2 diabetic patients, which can be linked to a number of parameters including for example age, body weight, and duration of diabetic symptoms^16–18^. In addition, different methods for the assessment of BCM can lead to varying results, depending on the imaging technique and targeting probe in use^19^. Imaging of the endocrine pancreas is indeed particularly difficult due to the fact that beta cells are enclosed in the islets of Langerhans that are scattered throughout the pancreas, forming relatively small cellular clusters having sizes ranging from about 50 to 500 µm in diameter under normal conditions^20^. Despite their very large number (between 3,000 and 5,000 islets in mice) they only represent about 1–2% of the entire pancreas volume^21^, challenging a comprehensive investigation of beta cells at the entire population level *in situ* in their particular location within the pancreas.

In the current study, we investigate changes in functional beta cell mass during the development of diabetes in the RIP-DTR mouse model. In these transgenic mice the diphtheria toxin receptor (DTR) is expressed under the rat insulin promoter and therefore selectively expressed in pancreatic beta cells^22^. Binding of a single molecule of diphtheria toxin (DT) to the DTR was shown to be sufficient to robustly inhibit protein synthesis and lead to cell death^23^, demonstrating the strong efficiency of this ligand-drug combination. Due to this potency only a single treatment of this mouse model with DT can cause a rapid near-total ablation of the pancreatic BCM^24^. Although the RIP-DTR mouse cannot be considered a model to describe changes in BCM occurring during the development of type 1 diabetes, for their clearly differing mechanisms of beta cell ablation, this model has been used for a number of studies ranging from pancreatic islet biology to transplantation^22,25–27^. A precise characterization of the kinetics and degree of functional beta cell mass decay is however lacking and would therefore be of high methodological relevance for any future research based on this mouse model.

In order to circumvent the inherent difficulty of assessing the decline in BCM both at high resolution and at the level of the entire islet population, we employed a novel combination of imaging tools. Optical projection tomography (OPT) imaging of the insulin-stained and tissue-cleared whole mount RIP-DTR pancreas was complemented with repeated confocal imaging of RIP-DTR islets transplanted into the anterior chamber of the eye (ACE). While OPT ensures accurate quantification of the total beta cell volume, islet number and distribution within the mouse pancreas^28^, the ACE model allows to monitor the precise kinetics of BCM decay by longitudinal assessment of a small number of islet grafts serving as ‘reporters’ for changes occurring in the pancreas^29^. Information obtained this way was further combined with various physiological and functional studies at different time points, leading to a complete appreciation of functional beta cell mass in the RIP-DTR mouse model.

## Results

### Kinetics of beta cell mass decay in the RIP-DTR mouse model

**Figure 1:**
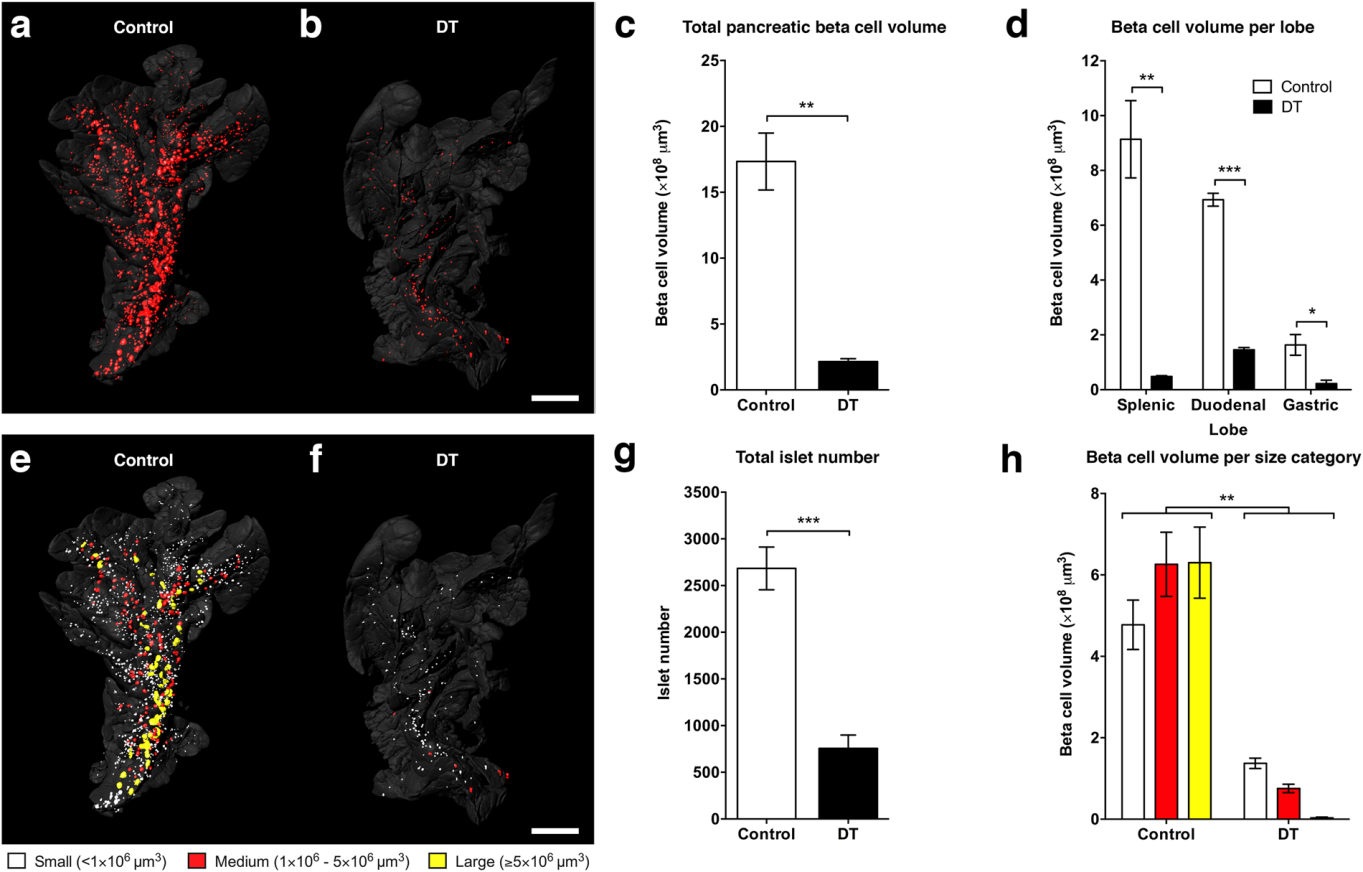
Optical projection tomography shows pancreatic beta cell loss in the RIP-DTR mouse. **(a**,**b**) Isosurface rendered OPT images of representative splenic lobes of the RIP-DTR mouse at 15 days after sham (**a**) or diphtheria toxin (**b**) treatment. Three-dimensional surface renderings are based on anti-insulin staining (red) and intrinsic autofluorescence portraying the anatomy (grey). (**c**) Graph of the average total pancreatic beta cell volume illustrating severe beta cell loss after diphtheria toxin treatment. (**d**) Graph showing that beta cell loss in the RIP-DTR pancreas is seen in all three lobes. (**e**,**f**) OPT images displaying pseudo-colours yellow (>5 × 10^6^ μm^3^), red (1–5 × 10^6^ μm^3^), and white (<1 × 10^6^ μm^3^) to indicate islets of different sizes in the splenic lobe of control (**e**) and RIP-DTR (**f**) pancreas. (**g**) Graph depicting the overall number of islets in the pancreas of sham or diphtheria toxin treated mice. (**h**) Graph showing the average cumulative beta cell volume of all islets within their respective size categories. Data presented as mean ± SE with n = 4 animals per group. Statistical significance indicated as ^*^P < 0.05, ^**^P < 0.01, ^***^P < 0.001. Scale bars = 2 mm.

To investigate the effect of DT on the degree of beta cell destruction at the entire pancreatic islet population level, a single dose of the toxin was administrated intraperitoneally to RIP-DTR mice. Fifteen days after treatment the pancreata were collected, cleared and stained using insulin antibodies. Samples were scanned by OPT and images were subsequently processed and analysed, allowing the identification of insulin-positive clusters defined as islets of Langerhans (Fig. 1a,b, Supplementary Movie 1). Quantification of the data shows that DT-treated animals have an average pancreatic beta cell volume corresponding to only ~10% of that of PBS-treated controls (Fig. 1a–c), demonstrating the high efficiency of the treatment. We next examined the OPT data to assess whether the degree of destruction was dependent on islet size and/or tomographic location within the pancreas, as has been shown for other mouse models of BCM decline^11^. Separate analysis of BCM in the splenic, duodenal, and gastric lobes demonstrates that beta cell destruction in the RIP-DTR mouse occurred similarly in all three pancreatic lobes, without any noticeable difference in spatial sensitivity within a lobe (Fig. 1d). When assessing islets in arbitrarily defined size categories (“small” <1.0 × 10^6^ μm^3^, “medium” 1.0–5.0 × 10^6^ μm^3^, and “large ≥5.0 × 10^6^ μm^3^), we determined that the number of islets present in each category decreased and that no large islets were remaining in DT-treated pancreata (Fig. 1e–g). The major contribution to the remaining total beta cell volume after DT treatment thus predominantly came from small islets (Fig. 1h). Similar analysis of pancreata at an intermediate time point after treatment confirmed that islet destruction occurred independently of their original size and location (Supplementary Fig. 1). Taken together, these results show that islets from different locations and size categories were equally sensitive to DT, and that there was a shift from islets in the large categories to the smaller categories.

**Figure 2:**
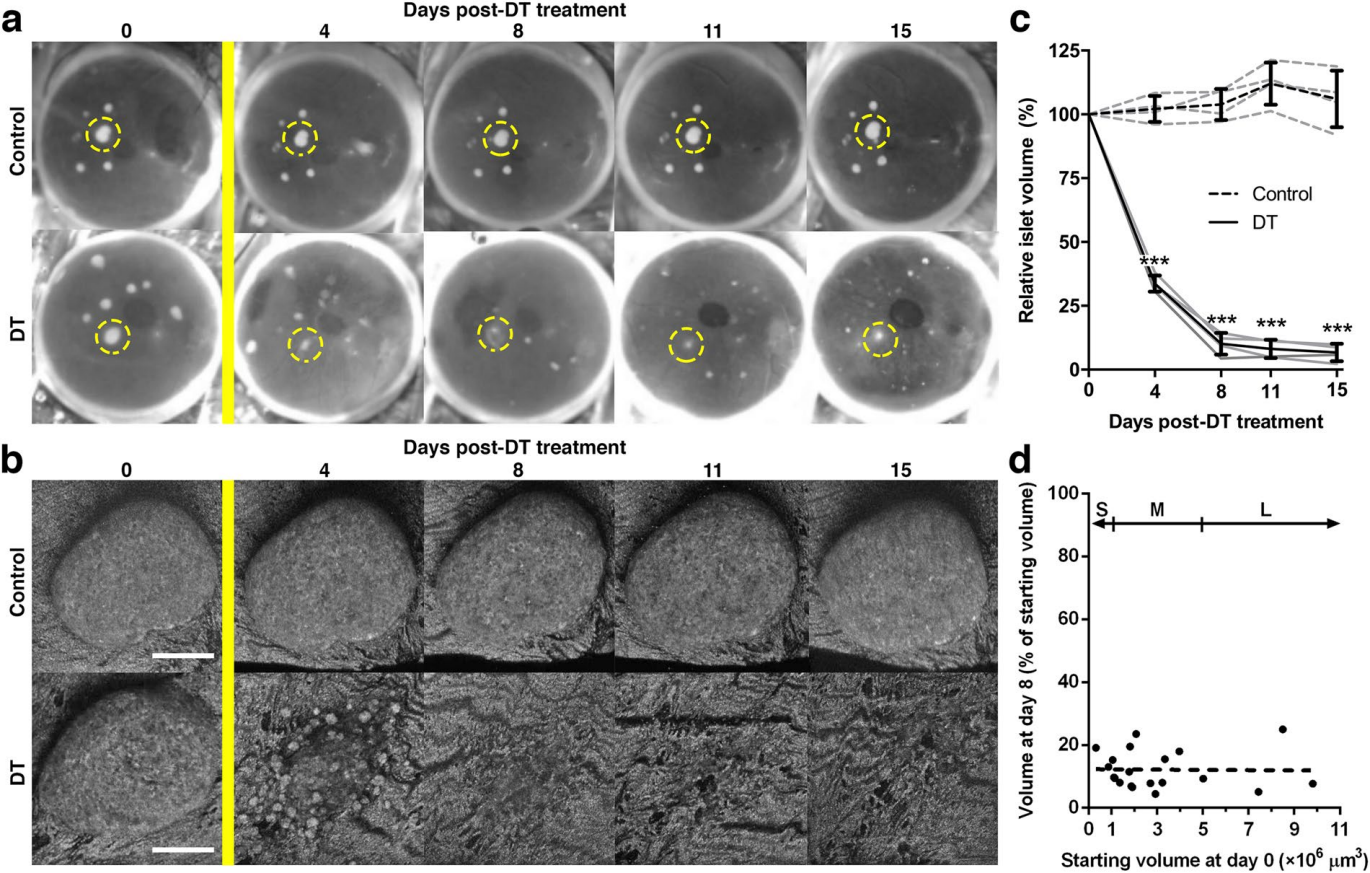
Repeated *in vivo* confocal imaging details the destruction of RIP-DTR islets over time. (**a**,**b**) Consecutively collected photographs (**a**) and confocal images (**b**) of engrafted RIP-DTR positive and negative islets in the eye of a diphtheria toxin-resistant control mouse. Yellow bar indicates diphtheria toxin treatment, dashed yellow circles in (**a**) show examples of individual islets followed over time in (**b**) using backscattered light to monitor morphological changes. (**c**) Graph showing the relative islet volume of RIP-DTR positive and negative islets in the anterior chamber of the eye over time following DT treatment. Islet volumes are shown averaged per mouse (grey lines, n = 4–11 islets per eye) and averaged per animal (black lines, n = 4 mice). (**d**) Relative decrease in islet volume (day 8) of individual RIP-DTR positive islets plotted against their initial starting volume (day 0). Linear regression analysis shows that no size-dependent sensitivity to DT-treatment exists (R^2^ < 0.001, n = 20 islets). Islet size categories as defined for OPT data analysis (“S” = small, “M” = medium, “L” = large, see Fig. 1) are displayed for comparison purposes. Data presented as mean ± SD. Statistical significance indicated as ^***^P < 0.001. Confocal images are shown as maximum intensity projections. Scale bars = 50 μm.

With the aim of increasing the temporal resolution in the assessment of BCM destruction kinetics in individual mice, we next turned to high resolution confocal imaging of RIP-DTR islets engrafted in the ACE. One month after transplantation, a time point at which islet revascularisation is complete (Supplementary Fig. 2), we monitored individual islet sizes before and after DT treatment. Longitudinal *in vivo* imaging showed that, while control islets maintained their size and morphology, RIP-DTR islets shrunk by losing cells and imploding over time after DT administration (Fig. 2a,b). The total islet volume of RIP-DTR islets in the ACE decreased progressively and, 15 days after treatment, showed a similar degree of destruction compared to that found in the pancreas. Markedly, the majority of the destruction occurred within the first four days after DT treatment (Fig. 2c). Plotting the decrease in volume of individual islets against the initial volume of these islets corroborates our findings from the OPT data, in that the destruction kinetics were independent of islet size (Fig. 2d).

### RIP-DTR mice display impaired glucose tolerance prior to onset of hyperglycemia

**Figure 3:**
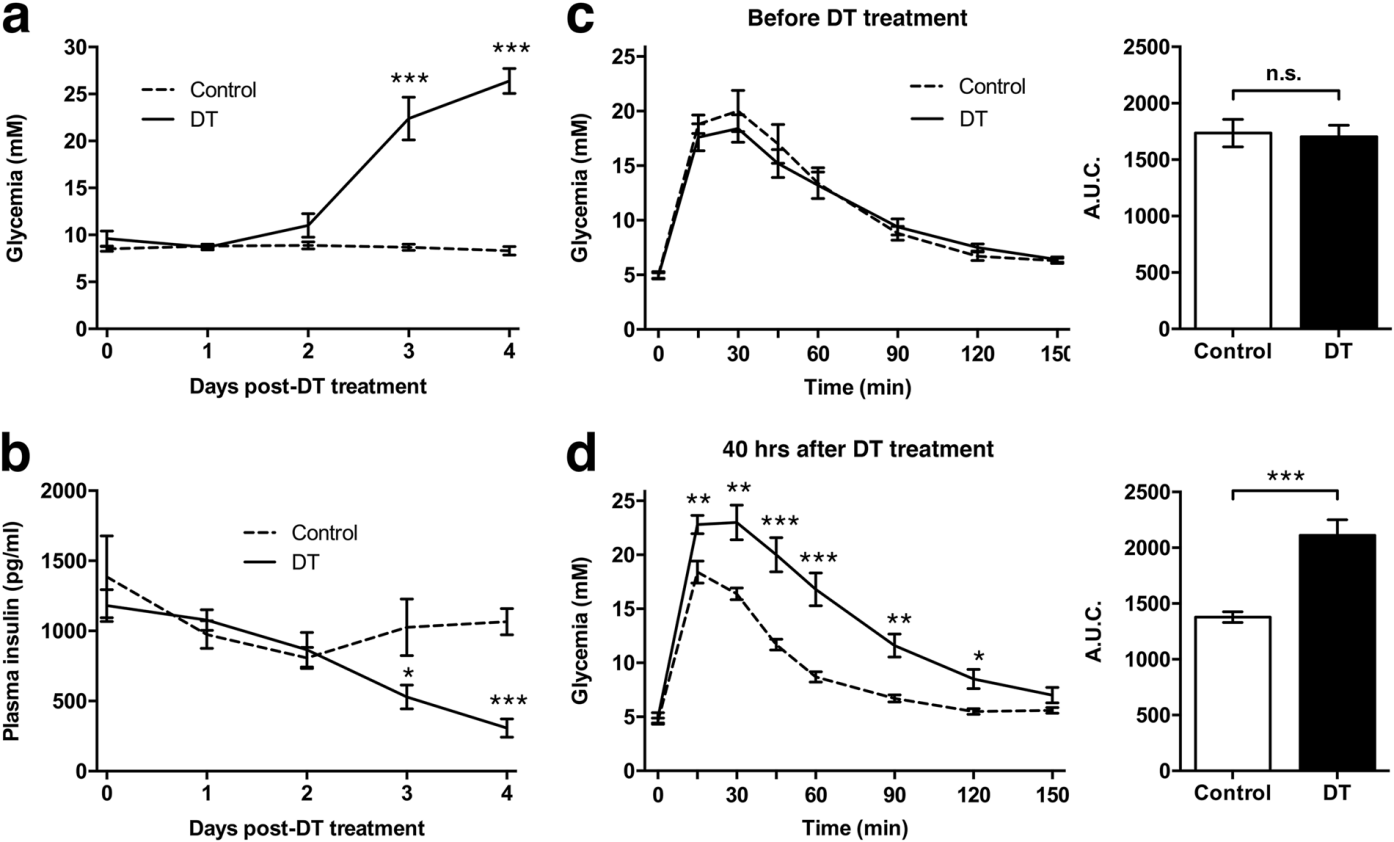
Metabolic features of the RIP-DTR mouse. (**a**,**b**) Unfasted blood glucose levels (**a**) and 4 hours fasted plasma insulin levels (**b**) measured daily following diphtheria toxin (solid line) or sham treatment (dashed line) of RIP-DTR mice. Control, n = 10 and DT, n = 5. (**c**,**d**) Graphs of glucose tolerance tests performed one week before and 40 hours after DT treatment of RIP-DTR positive and negative mice of mixed genders. Area under the curve (A.U.C.) was calculated, showing a significant difference 40 hours after treatment. Control, n = 9 and DT, n = 13. Data presented as mean ± SE. Statistical significance indicated as ^*^P < 0.05, ^**^P < 0.01, ^***^P < 0.001. n.s.: nonsignificant.

To complement our assessment of BCM destruction kinetics, we probed the effects of DT treatment on metabolic parameters over time. We found that RIP-DTR mice abruptly became hyperglycemic between two and three days after DT treatment (Fig. 3a). This observation correlated with a significant decrease in fasted plasma insulin levels after three days (Fig. 3b), whilst no change in food intake was observed (data not shown). Both RIP-DTR positive and negative mice exhibited normal glucose clearance before DT treatment (Fig. 3c). However, 40 hours post-DT treatment, when the mice were still normoglycemic, glucose tolerance in RIP-DTR positive mice was significantly impaired compared to controls (Fig. 3d).

### RIP-DTR islet function is impaired before substantial loss of islet mass

**Figure 4:**
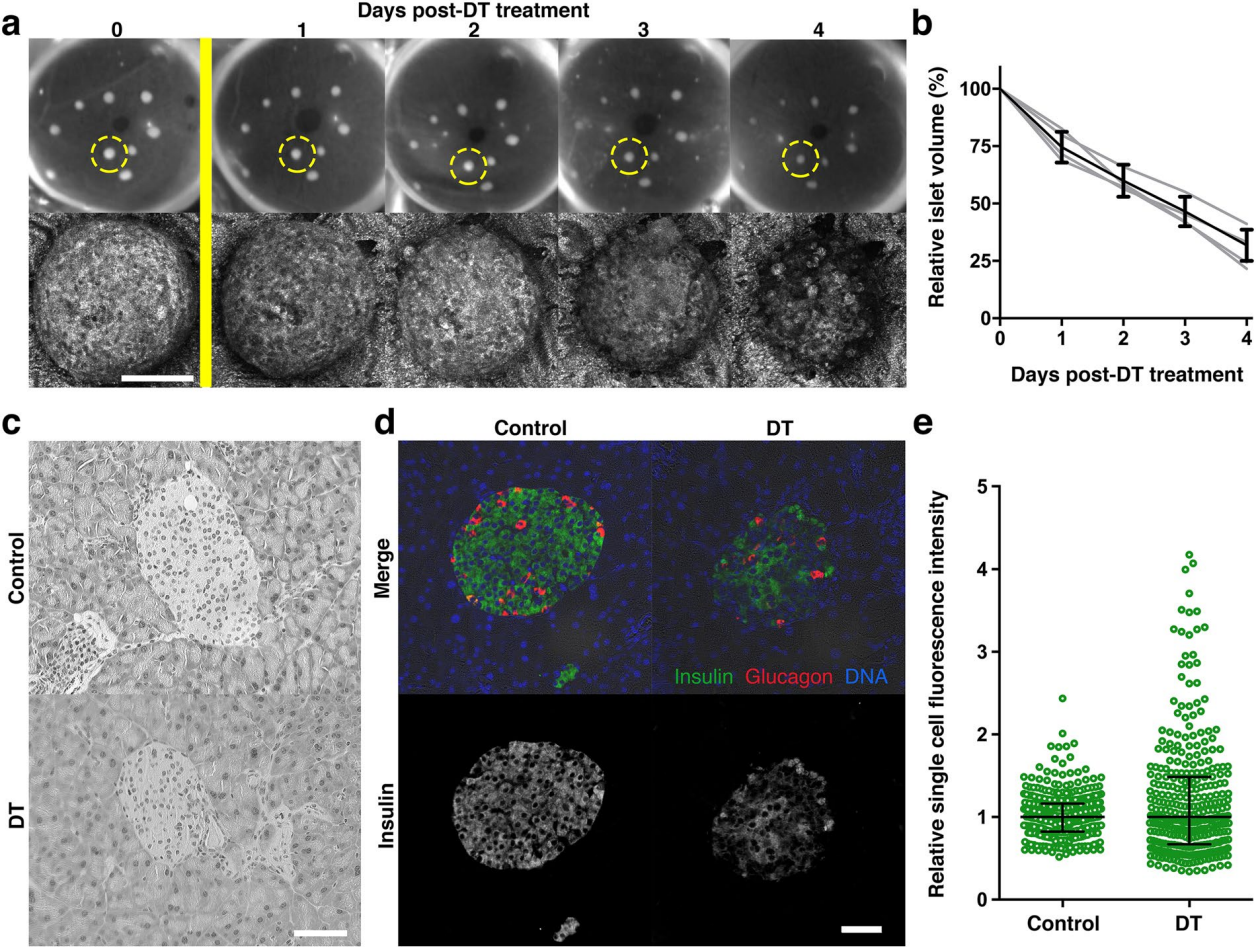
Changes in beta cell mass and insulin expression patterns during RIP-DTR islet destruction. **(a)** Photographs and confocal images of RIP-DTR islets engrafted in the anterior chamber of the eye of RIP-DTR mice over the course of four days following diphtheria toxin administration (yellow bar). Reflected light images of the encircled islet are shown over time. (**b**) Quantification of confocal images per animal (grey lines, n = 3–10 islets) and overall mean ± SD (black line, n = 4). Graph shows a decrease of relative islet volumes, representing a gradual beta cell destruction. **(c**–**e)** Histological examination of paraffin sections from the RIP-DTR pancreas collected two days after diphtheria toxin treatment depicts islets have a normal morphology based on haematoxylin and eosin staining (**c**) whereas anti-insulin staining (green) shows a large spread in the fluorescence intensity **(d**,**e)**. Graph shows individual insulin positive cells analysed (circles) and mean ± interquartile range (n = 3 mice, intensities are normalised per mouse to the average intensity). Confocal images are shown as maximum intensity projections. Scale bars = 50 μm.

To correlate the observed functional changes to the precise amount of BCM remaining at the time of impaired glucose handling, we examined the first four days of beta cell destruction in further detail by performing daily imaging of RIP-DTR islets engrafted in the ACE (Fig. 4a). Analysis of islet volumes over a four-day time period showed that beta cell loss was gradual, reaching ~70% decline in baseline volume (Fig. 4b). Hyperglycemia occurred when about half of the islet mass was lost (Figs 3a and 4b). At the 40-hour time point, right before hyperglycemia emerged, pancreatic islet morphology was still intact (Fig. 4c) and DT-treated islets stained positively for insulin (Fig. 4d), hence containing beta cells that contribute to the total BCM based on insulin staining. While islets from both groups stained positively for insulin, we found that the relative fluorescence from individual beta cells within the islet had a bigger spread in DT-treated mice (Fig. 4e). This lack of staining homogeneity is suggestive of alterations having occurred to protein expression levels or of changes in normal insulin secretory status at this time point. A compensatory over-secretion and depletion of insulin could for example result in a lower insulin staining in a subgroup of beta cells, whereas defects in insulin secretory mechanisms could explain the increased staining in others.

**Figure 5:**
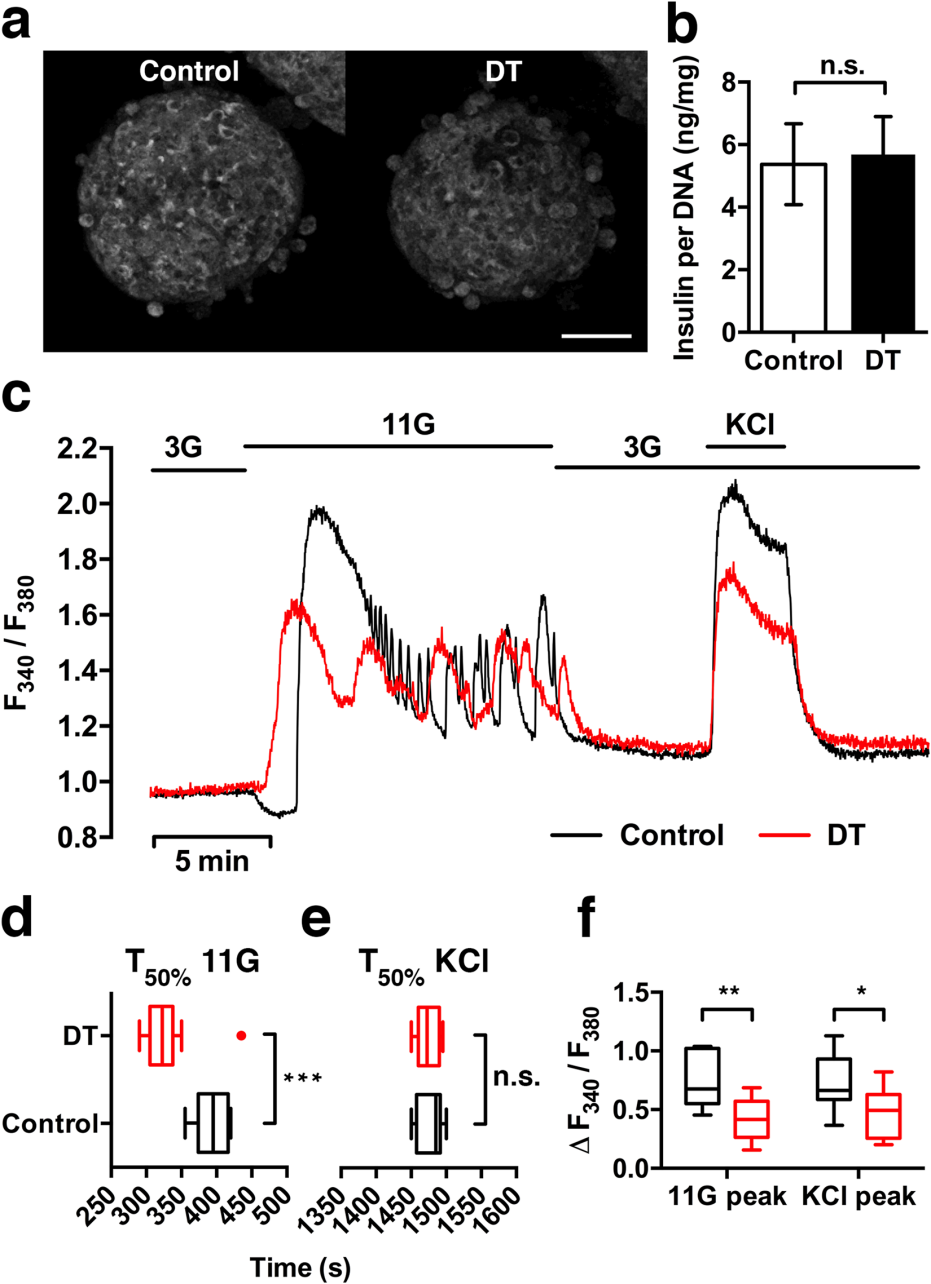
Insulin content and intracellular Ca^2+^ signalling of RIP-DTR islets one day after diphtheria toxin treatment. (**a**) Representative confocal images of isolated islets one day after DT or sham treatment of RIP-DTR mice. Reflected light signal shows that DT-treated islets have preserved insulin granule scattering properties and similar morphology compared to controls. (**b**) Quantification of whole islet insulin content analysed by ELISA and corrected for total DNA content (mean ± SE, n = 8–9 samples per condition). (**c**) Representative traces from [Ca^2+^]_i_ measurements of islets loaded with Fura-2 AM and perfused for 2,000 s with 3 or 11 mM glucose and 25 mM KCl. (**d**–**f**) Analysis of the [Ca^2+^]_i_ traces (n = 10–12 islets per condition) for response time to the stimulus (time to reach 50% of the peak) and relative increase of fluorescence (peak height). Whiskers represent tukey (**d)** or min/max values (**e**,**f**). Islets originated from 3 mice per condition. Statistical significance indicated as ^*^P < 0.05, ^**^P < 0.01, ^***^P < 0.001, n.s.: nonsignificant. Confocal images are shown as maximum intensity projections. Scale bar = 50 µm.

To further investigate to what degree functional capacity is maintained at a relatively early time point after DT-treatment, we next isolated islets from DT-treated RIP-DTR mice for *in vitro* assessments. One day after treatment, the morphology of islets from DT-treated RIP-DTR mice was comparable to that of islets from sham-treated mice (Fig. 5a). Reflected light imaging (indicative of the islet insulin secretory potential^30^, Fig. 5a), electron microscopy (Supplementary Fig. 3), and insulin content measurements (Fig. 5b) confirmed the presence of insulin containing granules in DT-treated islets. We investigated changes in cytoplasmic free Ca^2+^ concentration ([Ca^2+^]_i_) as an indicator of beta cell function. Islets from both DT- and sham-treated mice showed an increase in [Ca^2+^]_i_ in response to stimulation with 11 mM glucose and KCl, and a decrease in [Ca^2+^]_i_ when returning to 3 mM glucose. However, DT-treated islets showed a significant difference in the timing of response to high glucose (Fig. 5d), whereas no difference was seen upon membrane depolarization with the non-metabolic stimulus KCl (Fig. 5e), indicating that DT treatment caused an abnormal beta cell response to glucose. Furthermore, the amplitude of increased fluorescence signal relative to baseline was found to be lower in DT-treated islets compared to controls (Fig. 5f), which confirmed an impairment in [Ca^2+^]_i_ handling. Thus, despite normal morphology and insulin content, RIP-DTR islets had a diminished functional capacity one day after DT treatment, a time point at which there was no substantial loss in beta cell mass.

## Discussion

We have investigated changes in functional beta cell mass taking place in the RIP-DTR mouse after DT treatment. Cross-validation of *in vivo* confocal microscopy and *ex vivo* OPT data proves that both modalities are capable of capturing similar changes in BCM. Confocal microscopy provides high cellular and subcellular resolution images enabling detailed longitudinal analysis at the level of the individual islet^29,31^, however only a few islets can be monitored over time. Moreover, the sizes of transplanted islets are dependent on the isolation procedure and thus don’t reflect the entire spectrum of sizes represented in the pancreas^32^. OPT imaging lacks the resolution provided by confocal imaging, but on the other hand is superior in giving an understanding of changes occurring in the whole islet population *in situ* in the pancreas^11,15^, thus complementing confocal microscopy data.

Previous studies using the RIP-DTR mouse have already shown that DT can induce rapid and selective beta cell destruction^22,24^. A near-total (>99%) beta cell loss is obtained 15 days after three consecutive treatments with DT, based on pancreatic insulin content, insulin transcription levels, and insulin-stained pancreatic slices. This extreme beta cell loss was shown to lead to the conversion of alpha cells to beta cells^22^. Virostko and colleagues gave only a single dose of DT and found, using an *in vivo* bioluminescence approach and by measurements of pancreatic insulin content, an approximate 70% loss in BCM four days later^24^. In the current study, we used a single dose of DT to be able to monitor the kinetics of BCM decay at the temporal resolution of one day. At the time of DT administration all islet grafts were fully vascularised, allowing each beta cell to be in contact with blood vessels^33–35^ and thus ensuring a potential access to the toxin similar to that for islets *in situ* in the pancreas. Our data, based on both insulin labelling and morphometric analysis of confocal data, showed a 70% and 90% decrease in BCM at 4 and 15 days post-DT, respectively, and is thus comparable with previously published results indicating that the majority of beta cell destruction occurs within the first few days after treatment. Taken together, these data indicate a strong efficiency of DT destroying the beta cells in the RIP-DTR mouse, without excluding that there might still be a certain degree of dose-dependency in the RIP-DTR mouse in line with what was found in other DTR models^36^. The possibility to titrate beta cell destruction has been previously investigated by Matsuoka *et al*. in an alternative mouse model expressing DTR under two other beta cell specific promoters^37^. Although mainly based on glycemia measurements they found that even very low concentrations of DT lead to severe hyperglycemia, thereby challenging the notion of dose-dependency. Possibly, using our imaging approach, future studies might clarify the feasibility of titrating BCM in the RIP-DTR mouse model and hopefully delineate the precise dosages needed to achieve certain degrees of beta cell loss. This information would render this model a powerful alternative to treatments with agents such as streptozotocin and alloxan. The latter have been the primary methods of choice to achieve hyperglycemia and beta cell loss, but come with several side-effects as well as sex and strain differences that are likely absent using diphtheria toxin^37^.

Interestingly, and in contrast to what has previously been found in other models of beta cell loss, we report here that RIP-DTR islets are all equally sensitive to DT regardless of their size or spatial location in the pancreas. In the NOD mouse for instance, the large islets that comprise most of the total BCM resist initial immunological destruction^11^. A heterogeneous beta cell destruction pattern has also been observed in humans with insulin-dependent diabetes mellitus^38^. Although the mechanism of BCM decay in the RIP-DTR mouse does evidently not reflect that occurring in type 1 diabetes, the non-selective characteristics of DT-mediated destruction in this mouse model might prove to be a useful asset for the study of beta cell destruction-induced hyperglycemia and subsequent pathological consequences, potentially allowing a more precise control and titration of BCM in future studies. Indeed, in case of the RIP-DTR mouse model, beta cell destruction is not depending on factors such as inflammation or autoimmune attack that are more selective for specific subsets of pancreatic islets.

Inactivation of the eukaryotic elongation factor 2 in DTR-expressing cells is a rapid process occurring within an hour following DT administration^39,40^. This leads to the inhibition of protein synthesis, and eventually to cell death. It is however unclear at what time point after treatment cellular function is affected, or more precisely in our case, when beta cells stop secreting insulin in response to glucose. Using [Ca^2+^]_i_ as an indicator of islet function, we found diminished response amplitudes in the RIP-DTR islets upon high glucose and KCl stimulation. In accordance with a delayed glucose clearance this data suggest that DT-treated beta cells have an impaired glucose-stimulated insulin secretion, which might result in part from sub-optimal Ca^2+^ entry through voltage-gated Ca^2+^ channels. In agreement with previous studies on isolated islets^41,42^, we found that stimulation with glucose transiently reduces [Ca^2+^]_i_ before showing an increase in control islets. This so-called “dip” or “phase 0” response has been shown to reflect a normal sequestration of Ca^2+^ by the ER^41^. Interestingly, DT-treated RIP-DTR islets lack this transient decrease in [Ca^2+^]_i_, and as a consequence display a faster elevation of [Ca^2+^]_i_ following glucose stimulation. Although the exact reason for this observation has not been investigated in detail, our data suggest that DT-treated RIP-DTR islets have, among other things, disturbances in ER function that may result from an impairment in the sarco(endo)plasmic reticulum Ca^2+^ ATPase (SERCA) pump activity^41^. The absence of proper [Ca^2+^]_i_ buffering likely contributes to a precipitated cell death and subsequently to the decline in BCM observed a few days later.

Considering the fact that protein synthesis is completely inhibited in almost all beta cells shortly after DT administration, one can expect a drastic change in their function. It is therefore somewhat surprising that islets are still glucose responsive to a certain extent one day later. Similarly, although glucose tolerance was impaired two days after treatment, it is remarkable that beta cells are still able to secrete enough insulin to lower blood glucose levels, indicating that the functional capacity of the endocrine pancreas is partially maintained for a relatively long period of time after inhibition of protein synthesis.

In summary, our data illustrate that a combination of quantitative morphological and functional parameters is mandatory to assess the status of the endocrine pancreas in preclinical and clinical studies, in order to provide a more complete appreciation of disease progression and of the efficacy of various specific treatment strategies for diabetes.

## Methods

### Chemicals

All chemicals were from Sigma-Aldrich unless otherwise specified.

### Mouse model

Mice used in this study were bred in-house either by crossing heterozygous couples or by crossing RIP-DTR mice^22^ with C57Bl/6J (Charles River Laboratories, Germany), and genotyped prior to experiments. Control mice were either RIP-DTR negative mice or C57Bl/6 J mice, as indicated. Mice were kept in controlled temperature (22 ± 2 °C) conditions with a 12 h light/12 h dark cycle, and had free access to water and standard rodent chow diet. Mice were males between four and eight months of age unless otherwise specified. All animal experiments were performed in accordance with guidelines and regulations of Karolinska Institutet for care and use of animals in research and were approved by the Animal Ethics Committee at Karolinska Institutet.

### Diphtheria toxin treatment and physiological measurements

Treatment consisted of a single i.p. injection of 500 ng of diphtheria toxin in phosphate buffered saline (PBS). Blood glucose was measured using an Accu-Chek Aviva system (Roche, Bromma, Sweden). For the assessment of insulin levels, plasma was collected in EDTA-treated tubes (Sarstedt, Nümbrecht, Germany) and measured with alphaLISA (Perkin Elmer, Upplands Väsby, Sweden). Glucose tolerance tests were performed after 12 hours overnight fasting, by giving an i.p. injection of 2 g glucose per kg bodyweight. To avoid prolonged hyperglycemia, DT-treated RIP-DTR mice used for subsequent OPT imaging were transplanted with ~100–150 RIP-DTR negative islets into the ACE, normalising their blood glucose levels as shown previously for streptozotocin-treated mice^43^.

### Immunohistochemistry

5-µm-thick paraffin-embedded tissue sections were prepared and processed for imaging as previously described^29^. Staining was performed with haematoxylin and eosin, or using guinea pig anti-insulin antibody (1:1,000 dilution, DAKO A0564, Agilent Technologies, Kista, Sweden) followed by goat anti-guinea pig Alexa Fluor 488 secondary antibody (1:1,000 dilution, Thermo Fisher Scientific, Stockholm, Sweden) and mounted with ProLong Gold Antifade with DAPI (Thermo Fisher Scientific). Specificity of the primary antibody was confirmed by the lack of staining when solely using the secondary antibody. Images were acquired using a BD Pathway 855 system (BD Biosciences, San Jose, CA, USA).

### Optical projection tomography

Processing of whole mouse pancreata for OPT scanning was performed as described^44^. In brief, pancreata were dissected in cold PBS removing membranes and lymph nodes, fixed in freshly prepared 4% paraformaldehyde for 2–3 hours, and washed in cold PBS. Samples were stepwise transferred to 100% methanol, exposed to five freeze/thaw steps, bleached (to reduce endogenous tissue fluorescence) overnight in a mixture of MeOH (Thermo Fisher Scientific), DMSO and 15% H_2_O_2_ (at 2:3:1 ratio), and finally rehydrated into TBST (0.15 M NaCl, 0.1 M Tris pH 7.5, 0.1% Triton X-100). Organs were blocked overnight in blocking buffer containing TBST with 10% goat serum and 0.01% sodium azide, stained with a guinea-pig anti-insulin primary antibody (1:500, DAKO A0564, Agilent Technologies, Kista, Sweden) and a secondary Goat anti Guinea-Pig 594 Alexa (1:500, Thermo Fisher A11076), both for 48 hours. The splenic, gastric and duodenal lobe were separately embedded in a filtered low-melting agarose gel and transferred to a tissue clearing mixture of benzyl alcohol (Scharlab, Barcelona, Spain) and benzyl benzoate (Acros Organics, Geel, Belgium). While the sample was rotated a series of projection images were collected at 488 and 594 nm excitation with a Bioptonics 3001 OPT scanner (SkyScan, Kontich, Belgium) to visualise anatomy and islets, respectively. Raw projection images underwent enhancement by applying a contrast limited adaptive histogram (CLAHE) algorithm with a 64 × 64 tile size as described before^45^. Tomographic reconstructions, made with NRecon v1.6.9.18 software (SkyScan), were loaded into Imaris 7.7.0 (Bitplane, Belfast, UK) for beta cell volume quantification. Objects smaller than 20,000 µm^3^ were removed to avoid the inclusion of debris or artefacts in the quantification of islet sizes, setting a detection threshold corresponding to spherical cell clusters of ~34 µm in diameter.

### Transplantation and imaging of islets in the anterior chamber of the eye

Islets were isolated via ductal injection of 1 mg/ml collagenase type A or P (Sigma) in Hanks’ Balanced Salt Solution (HBSS) supplemented with HEPES (pH 7.4), handpicked in HBSS containing 0.5% BSA, and cultured overnight in RPMI-1640 containing Pen/Strep and L-Glutamine (Thermo Fisher Scientific), with 10% fetal bovine serum. About 5–10 islets were transplanted into the anterior chamber of the eye of mice under isoflurane anaesthesia (Baxter, Kista, Sweden) as described previously^46^. The cornea was perforated with a 23 G needle to create a small hole through which islets could be introduced with a glass cannula connected via tubing to a Hamilton syringe. Post-operative analgesia was provided by s.c. injection of 2 μg Temgesic (RB Pharmaceuticals Limited, Slough, UK). Starting one month after transplantation (to allow for complete vascularisation of the islet grafts^43^), overview images of the transplanted eyes were obtained using a digital camera connected to a Leica M60 stereomicroscope, and high resolution imaging of the islet grafts were obtained by confocal microscopy using a Leica SP5 system with 25x objective (Leica Microsystems, Wetzlar, Germany). Viscotears (Laboratoires Théa, Clermont-Farrand, France) was used as an immersion medium between the lens and the mouse eye. Z-stacks of 4 μm thickness were acquired of every islet using backscattered light with excitation and collection at a wavelength of 633 nm^30^. To visualise islet vascularisation, 100 μl of a solution containing 2.5 mg/ml of 500-kDa FITC-labeled dextran (Invitrogen) was injected intravenously prior to fluorescence imaging using a 496-nm excitation wavelength. Confocal image stacks were analysed using Fiji^47^ 1.50d with the plugin Interactive Stack Rotation. The islet volume was extrapolated from projected area and perpendicular z-depth^30^.

### Structural and functional assessment of islets in vitro

RIP-DTR mice were subjected to DT or sham treatment as mentioned. Two hours after treatment, islets were isolated and cultured overnight. One day post-treatment, islets were used for structural and functional assessments. Insulin content was determined from groups of eight islets lysed in the protein extraction reagent M-PER (Thermo Fisher Scientific), using AlphaLISA (Perkin Elmer). Values were normalised to DNA content as determined using Quant-iT Picogreen dsDNA kit (Thermo Fisher Scientific). Islets for electron microscopy were fixed in 2.5% glutaraldehyde +1% paraformaldehyde in 0.1 M phosphate buffer, pH 7.4 at room temperature for 30 min and stored at 4 °C for processing as described^30^. Islets for [Ca^2+^]_i_ measurements were loaded for one hour with 2 μM Fura-2AM (Thermo Fisher Scientific) in a buffered solution (pH 7.4, containing 125 mM NaCl, 5.9 mM KCl, 2.6 mM CaCl_2_, 1.2 mM MgCl_2_, 25 mM Hepes, and 0.1% BSA) supplemented with 3 mM glucose. Islets were attached to a cover slip using PuraMatrix Hydrogel (BD Biosciences) and fluorescence was recorded using an inverted epifluorescence Axiovert 135 microscope (Zeiss, Jena, Germany) connected to a SPEX Industries Fluorolog spectrofluorometer for dual-wavelength excitation fluorimetry. Islets were perfused at 37 °C with the buffered solution supplemented with either 3 mM glucose, 11 mM glucose, or 3 mM glucose + 25 mM KCl, and simultaneously excited at 340 and 380 nm. Fluorescence emission was recorded after 510/40 nm bandpass filter every second for both excitation wavelengths, and the ratio of these two signals was calculated for normalisation.

### Statistical analysis

Data were processed with Excel (Microsoft, Redmond, WA, USA) and Prism version 6.0 (GraphPad Software, La Jolla, CA, USA), and are presented as mean ± SE or mean ± SD as indicated. Student’s unpaired t-tests were used for statistical analysis. P-values < 0.05 were considered statistically significant.

### Data availability

The data generated in this study are available from the corresponding author upon request.

## Electronic supplementary material

Supplementary Movie 1

Supplementary Figures 1-3
