# Supplementary material for: Kinetics of functional beta cell mass decay in a diphtheria toxin receptor mouse model of diabetes
Source: Sci Rep. 2017 Sep 29;7:12440. doi: 10.1038/s41598-017-12124-w (PMC5622115; doi:10.1038/s41598-017-12124-w)
Supplement: Supplementary file 2 — Supplementary Figures 1-3 [file 41598_2017_12124_MOESM2_ESM.pdf]

# Supplementary information

## Kinetics of functional beta cell mass decay in a diphtheria toxin receptor mouse model of diabetes

Pim P. van Krieken, Andrea Dicker, Maria Eriksson, Pedro L. Herrera, Ulf Ahlgren, *Per-Olof Berggren, Erwin Ilegems

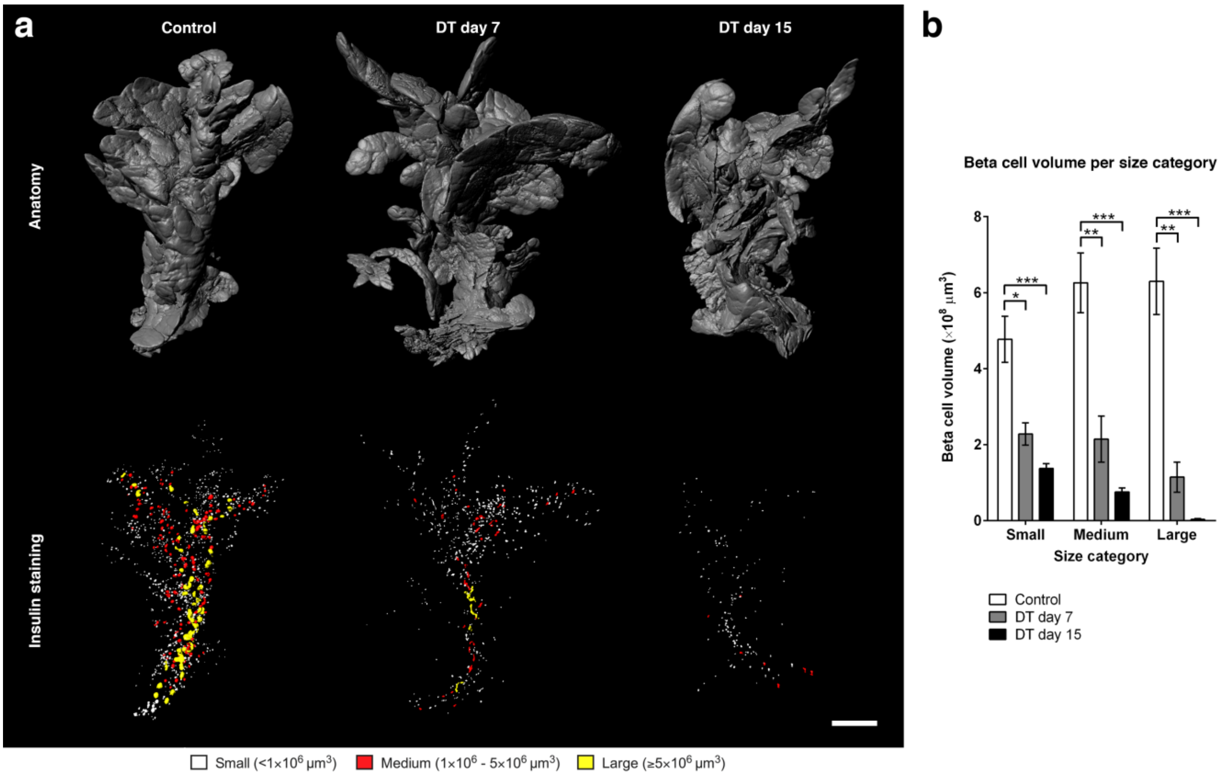


**Supplementary Figure 1.** Beta cell destruction pattern in the RIP-DTR pancreas. (**a**) Three-dimensional surface rendered OPT images of representative splenic lobes of the pancreas of RIP-DTR mice. Depicted are the anatomy based on intrinsic autofluorescence and anti-insulin staining showing islets pseudo-coloured to represent three size categories. Pancreata were collected 7 or 15 days after administration of a triple or single dose of 500 ng DT, respectively. (**b**) Graph illustrating the total pancreatic beta cell volume per islet size category for pancreata collected 7 or 15 days after DT treatment (n = 3-4). The progressive decrease in size of islets initially located in the core and peripheral regions of the pancreas demonstrate that islets from different sizes are equally sensitive to diphtheria toxin, leading to a gradual shift from islets in the large category to the medium and small categories. Statistical significance compared to sham treated controls collected at day 15 is indicated as $^{*}P < 0.05$, $^{**}P < 0.01$, $^{***}P < 0.001$. Scale bar = 2 mm.

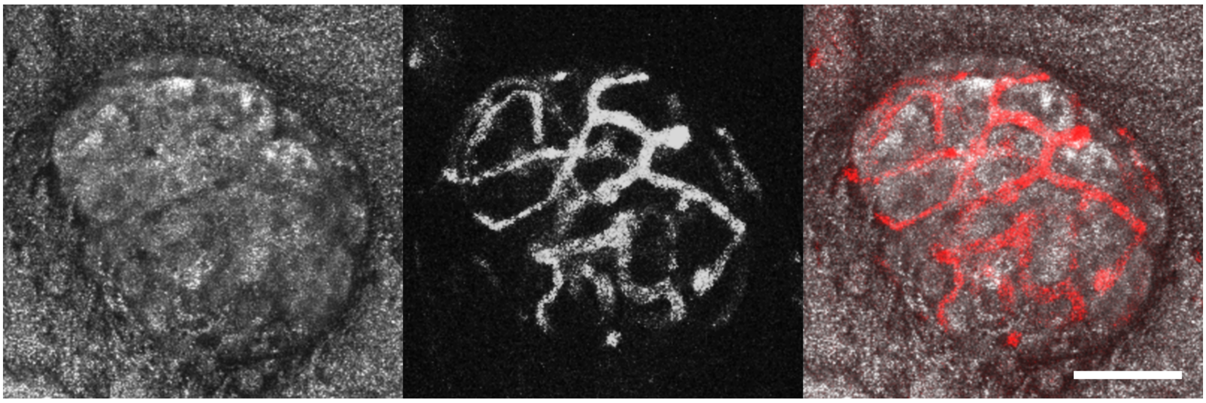

**Supplementary Figure 2.** Vasculature of engrafted RIP-DTR islets in the ACE. *In vivo* confocal images (maximum intensity projections) of a representative RIP-DTR islet one month after transplantation before DT treatment show full vascularisation enabling DT to efficiently reach beta cells within the islet graft. Panels (left to right) display islet morphology by reflected light imaging, blood vessels by injected fluorescently labelled dextran, and merged image. Scale bar = 50 μm.

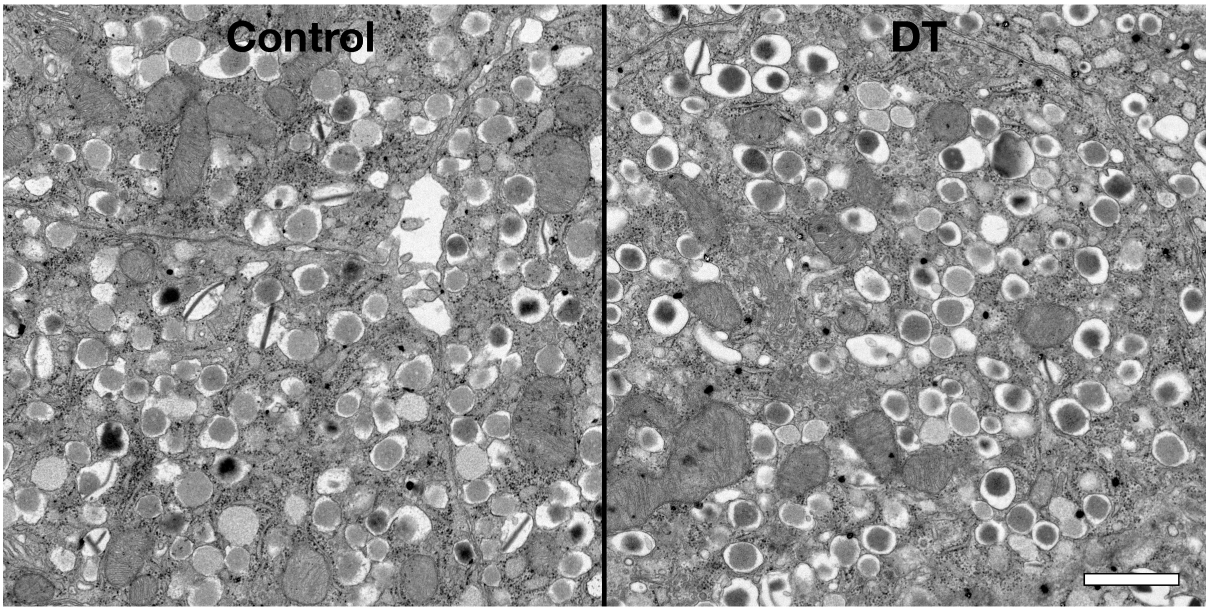


**Supplementary Figure 3.** Ultrastructure of pancreatic beta cells after diphtheria toxin (DT) treatment. One day after DT or sham treatment, islets from RIP-DTR mice were processed and imaged by electron microscopy. No apparent differences were observed in terms of insulin granule content, morphology, and density in islet beta cells after DT treatment, supportive of unaltered insulin content and secretory potential. Images are representative. Scale bar = 1 μm.
